# Enhanced anti-HCV activity of interferon alpha 17 subtype

**DOI:** 10.1186/1743-422X-6-70

**Published:** 2009-06-03

**Authors:** Aurelie Dubois, Catherine François, Veronique Descamps, Carole Fournier, Czeslaw Wychowski, Jean Dubuisson, Sandrine Castelain, Gilles Duverlie

**Affiliations:** 1 Virology Laboratory-Amiens University Medical Centre, France; 2 CNRS-UMR 8161, Lille Institute of Biology, Lille, France

## Abstract

**Background:**

Pegylated interferon alpha 2 (a or b) plus ribavirin is the most effective treatment of chronic hepatitis C but a large proportion of patients do not respond to therapy. So, it is interesting to improve the treatment efficacy. Interferon alpha is a type I interferon composed of 12 different subtypes. Each subtype signals by the Jak-Stat pathway but modulations in the antiviral activity was previously described.

**Methods:**

Using the hepatitis C virus (HCV) culture system, we have tested the anti-HCV activity of each interferon alpha subtypes. We have analyzed the effect of each subtype on the HCV multiplication and the cell-signaling pathway for some subtypes.

**Results:**

There were divergent effects of IFN alpha subtypes against HCV. We have found that IFN alpha 17 was three times more efficient than IFN alpha 2a on HCV. This efficiency was related to a stronger stimulation of the Jak-Stat pathway.

**Conclusion:**

We suggest that IFN α17 should be tested therapeutically with a view to improving treatment efficacy.

## Background

The hepatitis C virus (HCV) is one of the main known causes of liver diseases such as cirrhosis and hepatocellular carcinoma (HCC) [1,2]. Infection with HCV is a major public health problem; it has been estimated that 3% of the world's population is chronically infected. Indeed, in many countries, HCV is the most common cause for liver transplantation [3,4]. Current therapy is based on pegylated interferon alpha 2a or 2b, in combination with ribavirin [3]. Nevertheless, combination therapy is not fully effective (with only approximately 55% of patients showing a sustained virological response) and its frequent side-effects reduce health-related quality of life in many patients [5]. Improvement of HCV therapy implies (i) to gain a better understanding of the mechanism of action of current treatments and (ii) to develop novel anti-HCV molecules [6,7]. Recent data concerning new molecules (such as anti-polymerases and anti-proteases) used in monotherapy have shown that escape mutants are rapidly selected for. Hence, administering these molecules in combination with interferon may be one way of improving treatment efficacy [8-11].

Interferon alpha (IFN-α) is a cytokine that has many biological properties; it is antiviral and antiproliferative and stimulates cytotoxic activity in a variety of immune system cells [12]. Interferon alpha is a member of the type I interferon family, comprising cytokines that bind to the same receptor (the interferon α/β receptor, IFNAR) to initiate a signaling response [13]. Several subtypes of IFN-α (12 proteins encoding by 14 genes) and many allelic variants have been described. Interferon alpha subtypes exhibit a very high degree of amino-acid similarity (over 75%) but the reason for the existence of so many distinct proteins is still unknown [12,13]. Although each subtype displays a unique activity profile [12,14], only IFN-α2a and IFN-α2b subtypes are currently used for the treatment of chronic HCV infection. After binding to the IFNAR, IFN-α signals mainly through the Jak-Stat pathway. The Janus kinases Jak-1 and Tyk-2 are then phosphorylated and, in turn, phosphorylate STAT proteins, which multimerize and associate with IRF-9 to form ISGF3 (interferon-stimulated gene factor 3). This complex translocates to the nucleus and targets the ISRE (interferon-stimulated response element) sequences present within the promoters of interferon-stimulated genes (ISGs) coding for (amongst others) a number of antiviral proteins, including the well-characterized antiviral PKR protein (double-stranded RNA-dependent protein kinase), 2'-5' oligoadenylate synthetase (2-5OAS) and MxA [15].

Several studies have focused on the differing degrees of antiviral action produced by the various IFN-α subtypes. Foster *et al*. have shown that IFN-α8 was the most potent subtype in various human cell lines infected with murine encephalomyocarditis virus (EMCV), whereas IFN-α1 had very little antiviral effect in the same system [16]. These results were confirmed by Yamamoto *et al*. in human hepatic cell lines infected by vesicular stomatitis virus (VSV) [17]. The antiviral effects of IFN-α subtypes on HCV has also been studied using subgenomic replicons [18]. Koyama *et al*. have demonstrated that the various IFN-α subtypes differ in terms of their anti-HCV actions and that IFN-α8 was the most effective inhibitor of intracellular HCV replication. These authors' results suggest that this differential effect may be exerted through JAK-STAT-independent pathways [19].

The recently developed HCV cell culture (HCVcc) system uses a JFH-1 genotype 2a strain of HCV and enables investigation of the overall viral life cycle [20]. In the present work, we used this system to determine the anti-HCV activity of twelve recombinant IFN-α subtypes. The antiviral action of each subtype was compared with that of IFN-α2a (i.e. the subtype used in therapy) by measuring intracellular viral replication and the production of infectious virions. Having found that IFN-α17 displayed the highest anti-HCV activity, we then explored the transduction pathways which could explain this heightened ability. Our results show that IFN-α17's anti-HCV activity may be accounted for by stronger activation of the JAK-STAT pathway and thus higher antiviral protein expression levels.

## Methods

### Cell culture and viral infection

Huh7 human hepatoma cells were cultured in Dulbecco's modified Eagle's medium (DMEM) (Jacques Boy, Reims, France) supplemented with 10% fetal calf serum and maintained in 5% CO_2 _at 37°C. JFH-1 viral stock preparation and titration were performed exactly as described previously [21].

### Recombinant interferon alpha subtypes

All the recombinant IFN-α subtypes (α1, α2a, α4, α5, α6, α7, α8, α10, α14, α16, α17 and α21) were obtained from the human IFN sampler (PBL Biomedical Laboratories, Piscataway, NJ). It includes 2.10^5 ^units/mL of each subtype of IFN-α. The concentration in pg/mL was taken into account for each subtype. The interferon subtypes were quantified using the VSV challenge assay on MDBK cells, as supplied by the manufacturer (Table 1).

**Table 1:** Specific activity of each interferon alpha subtype.

IFN-α subtype	Specific activity (IU/mg)	IFN-α subtype	Specific activity (IU/mg)
IFN-α 1 (D)	7.5.10^7^	IFN-α 8 (B2)	4.95.10^8^
IFN-α 2a (A)	3.85.10^8^	IFN-α 10 (C)	2.31.10^8^
IFN-α 4b	1.8.10^8^	IFN-α 14 (H2)	1.05.10^8^
IFN-α 5 (G)	2.33.10^8^	IFN-α 16 (WA)	2.4.10^8^
IFN-α 6 (K)	1.48.10^8^	IFN-α 17 (I)	1.4.10^8^
IFN-α 7 (J1)	2.56.10^8^	IFN-α 21 (F)	6.3.10^8^

All specific activities were given by the manufacturer and were titrated by using a cytopathic effect inhibition assay. The units are determined with respect to international reference standard for human interferon alpha α provided by the National Institutes of Health. Units of activity were measured in bovine MDBK cells with vesicular stomatitis virus (VSV).

### Determination of anti-HCV efficacy

Huh7 cells were infected with JFH-1 at a multiplicity of infection (MOI) of 0.001. After three weeks of infection, chronically infected cells were seeded in 24-well plates at a density of 70,000 cells/well in medium (DMEM supplemented with 10% fetal calf serum) containing different IFN-α subtypes at a concentration of 260 pg/mL. After 48 hours of incubation at 37°C, the supernatants were harvested and the virus yield was measured using a focus-forming unit (FFU) assay. The cells were washed twice with phosphate-buffered saline (PBS) and then trypsinized in order to perform HCV RNA quantification.

### Intracellular HCV RNA quantification

Total RNA was extracted from the cells using the RNeasy Mini kit ("Animal cell spin" protocol) from Qiagen (Courtaboeuf, France), according to the manufacturer's instructions. HCV RNA quantification was performed with a real-time RT-PCR assay, as previously described [22]. At the same time, β-actin RNA was quantified by including 1.25 *μ*L of human β-actin mix (Applied Biosystems, Coutaboeuf, France) in the PCR mix reaction instead of the HCV primers and probe. Each IFN-α subtype's inhibitory activity on the JFH-1 strain was calculated by using β-actin gene as a housekeeping gene and applying the comparative C_t _method, as previously described [23].

### Viral yield assay (FFUs)

35,000 cells were seeded into 24-well plates and infected with the supernatants at different dilutions (1 to 10^-2^). After 6 hours of incubation, the medium was replaced with fresh medium (DMEM supplemented with 10% fetal calf serum). After 3 days, an immuno-peroxydase reaction was performed as previously described [21]. Focus-forming units were counted for each dilution and normalized to 1 mL

### IFN subtype IC_50 _determination

50,000 Huh7 cells were seeded into 24-well plates and infected with 200 *μ*L of the JFH-1 strain (MOI = 0.1) at different dilutions (10^-2 ^to 10^-4^). After 18 hours of incubation, the medium was replaced by fresh medium containing IFN-α subtypes at different concentrations (0, 1.3, 2.6, 5.2, 13 and 26 pg/mL). After 2 days of culture, FFUs were quantified, as previously described. The concentration that inhibited 50% of the yield (i.e. the IC_50_) was calculated for each condition.

### Interferon-stimulated response element luciferase reporter assay

Huh7 cells were seeded into 96-well plates at a density of 20,000 cells per well. The following day, the culture medium was replaced by fresh medium. Fours hour later, each well was transfected with 250 ng of the pMx-GFP-luc plasmid by using the calcium phosphate precipitation technique (CalPhos Mammalian Transfection kit, Clontech, Saint-Germain en Laye, France) according to the manufacturer's instructions. The plasmid had been constructed by inserting the human MxA promoter [24] into the pEGFPLuc vector (Clontech). On the following day, IFNs were applied to the culture medium at various concentrations (0, 26 and 260 pg/mL). After 18 hours, cells were lysed and luciferase activity was quantified using the Luciferase Assay System (Promega, Charbonnieres-les-bains, France) in a luminometer (Lumat, Berthold, Thoiry, France).

### Transcriptome studies using a low-density array

300,000 chronically-infected Huh7 cells were seeded into 6-well plates and incubated with IFN-α2a, IFN-α17 or IFN-α1 (at 260 pg/mL in all cases). After 48 hours, total RNA was extracted from the cells and reverse-transcribed into cDNA, as previously described. The TaqMan^® ^Low Density Array (TLDA) is a 384-well microfluidic card that enables the performance of 384 simultaneous real-time PCRs. Each 2-*μ*l well contains specific, user-defined primers and probes capable of detecting a single gene. In the present study, the TLDA card was configured into two 96-gene sets which enabled analysis of gene expression in 2 different conditions. These genes (chosen on the basis of the literature) were present in duplicate and were all expressed under the control of the ISRE [25]. One hundred ng of each cDNA sample were mixed with an appropriate buffer (TaqMan Universal PCR Master Mix from Applied Biosystems), and was transferred into a loading port on the TLDA card. The card was then sealed and PCR amplification was performed using an Applied Biosystems Prism 7900HT sequence detection system. The ΔΔCt method was used for analysis after normalization to β-actine expression.

### Western blot analysis

IFNs were added to chronically-infected Huh7 cells at increasing concentrations (0.26, 2.6, 26 and 260 pg/ml) for either 30 minutes or 24 hours. At the indicated time point, cells were washed twice with cold PBS, harvested and then lysed using a buffer (1% NP40, 10% glycerol, 50 mM Tris-HCl pH 7.5, 150 mM NaCl and 0.5 mM PMSF) containing a phosphatase inhibitor cocktail (Sigma Aldrich, France) diluted to 1:100. Total cellular extracts were separated by SDS-PAGE electrophoresis and transferred to nitrocellulose membranes. The membranes were then incubated overnight at 4°C with the primary antibodies. The blots were developed with the chemiluminescence (ECL) system (GE Healthcare) using specific peroxydase conjugated anti IgG (GE Healthcare, Saclay, France) antibodies. The anti-MxA monoclonal antibody (Mab) was a gift from Dr I. Julkunen (Department of Viral Diseases and Immunology, National Public Health Institute, Helsinki, Finland). Anti-Stat1 and anti-PStat1(Tyr701) antibodies were purchased from Cell Signaling Technology and anti-β actin (C4) Mab was purchased from Santa Cruz Biotechnology (Tebu Bio, Le Perray en Yvelines, France).

### Statistical analysis

Statistical data analyses were performed using Student's T test. P-values of 0.05 or less were considered to be significant.

## Results

### Antiviral activity of different IFNα subtypes on chronic HCV replication and multiplication

**Figure 1:**
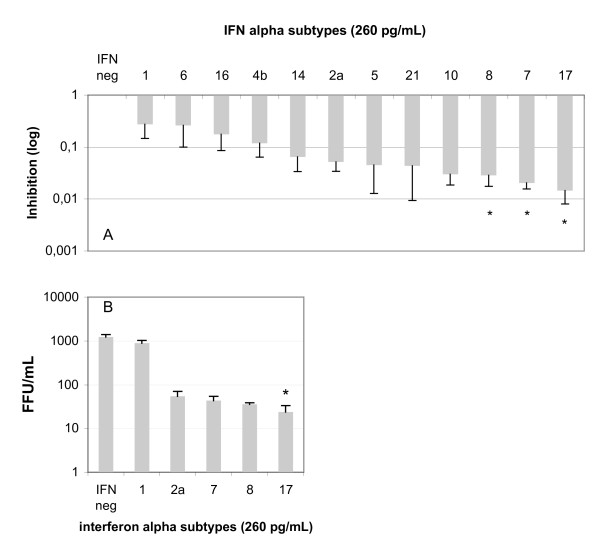
**Inhibition of HCV replication and multiplication by different IFN-α subtypes**. **A**: Total RNA from Huh7-infected cells cultured with 260 pg/mL of different IFN α subtypes was used to quantify HCV intracellular RNA, as described in the Materials and methods. The inhibition was calculated by comparing the results to infected Huh7 cells in the absence of interferon. An asterisk indicates represents the IFN subtypes that were significantly more potent against HCV than IFN-α2a. **B**: Quantification of HCV multiplication by measuring the viral yield in the supernatant. The results were expressed in FFU/mL on a semi-logarithmic scale. An asterisk indicates the IFN subtypes that were significantly more potent against HCV than IFN-α2a. The results correspond to the mean of four independent experiments.

Each IFN-α subtype exhibits a characteristic antiviral profile. Hence, we compared the respective anti-HCV activities of IFN-α subtypes obtained from the human IFN sampler. Two parameters were considered. Firstly, viral replication after treatment of infected Huh7 cells with 260 pg/mL of each IFN-α was measured by quantifying intracellular HCV RNA. The fold inhibition was then calculated, as described in the Materials and Methods section. As shown in Figure 1a, all IFN-α subtypes inhibited HCV replication (p < 0.05). A classification of the anti-HCV activity of IFN-α subtypes was carried out by comparing the values with the fold inhibition displayed by IFN-α2a. Three subtypes appeared to have greater activity against HCV: IFN-α17 (p < 0.001 versus IFN-α2a), -α7 (p < 0.005 versus IFN-α2a) and -α8 (p < 0.005 versus IFN-α2a). The other subtypes (notably IFN-α1) had modest effects on HCV replication.

Secondly, HCV multiplication was measured using the FFU technique. As shown in Figure 1b, only IFN-α17 was significantly most potent than IFN-α2a, and the results showed a correlation between the activity of IFN-α subtypes on viral replication and that on viral production. However, IFN-α17 stood out due to its major anti-HCV activity.

### Determination of the inhibitory concentration 50% (IC_50_) for IFN-α subtypes 2, 17 and 1

**Figure 2:**
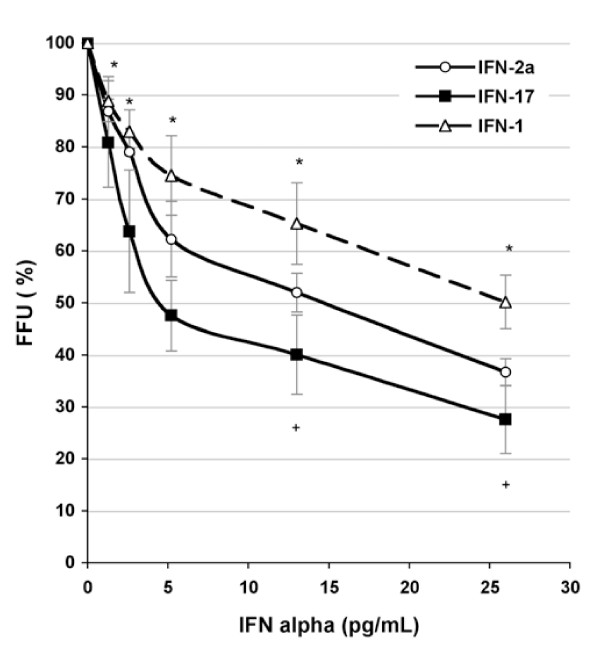
**Determination of the IC50% for three IFN-α subtypes**. Huh7-infected cells were cultured with or without different concentrations (in pg/mL) of IFN-α2a (open circles), IFN-α17 (closed squares) and IFN-α1 (open triangle). The virus yield was determined using the FFU method. The results represent the mean of four independent experiments. The p values were < 0.05 at all concentrations for the IFN-α2a vs. IFN-α17 comparison (*) and at 12.5 and 25 pg/mL for the IFN-α2a vs. IFN-α1 comparison (+).

In order to confirm the above results and analyze the action of certain subtypes, we determined the IC_50 _(the concentration of IFN-α required to decrease the production of infectious virions by half) for IFN-α2a, α17 and α1. Huh7 cells were infected with HCV-JFH1 and various concentrations of IFNs were added on the following day. Viral yields were then measured using an FFU assay and viral titers were expressed as a percentage relative to the IFN-free control well. As shown in Figure 2, the IC_50 _values for IFN-α2a and IFN-α17 were 14.6 pg/mL and 4.8 pg/mL, respectively. This was equivalent to about a 3-fold enhancement in activity. However, IFN-α1 had poor anti-HCV activity because a concentration of over 25 pg/mL was required to obtain the same degree of antiviral action as with IFN-α2a. The greater anti-HCV activity of IFN-α17 was thus confirmed.

### Stimulation of the ISRE-dependent gene by different IFN-α subtypes

**Figure 3:**
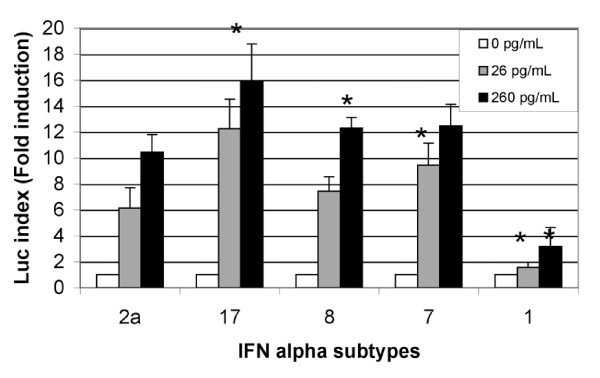
**Stimulation of the Mx promoter by the different IFN-α subtypes**. Huh7 cells were transfected with a plasmid containing the luciferase reporter gene under the control of the human Mx promoter. After stimulation by IFN-α2a, 17, 8, 7 and 1, luciferase was quantified. The results correspond to the ratio between the value obtained with IFN-α and the value obtained in the absence of interferon and were the mean of two independent experiments in triplicate. An asterisk indicates the values that were significantly different from those for IFN-α2a at the same concentration.

Two IFN-α subtypes stood out as a result of the antiviral activity studies: IFN-α17 and IFN-α1. The former was particularly potent against HCV and the latter had very low activity. We thus sought to explore the antiviral mechanism of each IFN-α subtype. Induction of the Mx promoter (mainly activated by ISRE elements) was first studied. As shown in Figure 3, all the tested IFNs subtypes activated the Mx promoter (via the Jak-Stat pathway) but to differing extents. The Mx promoter was notably less activated by IFN-α1 than by the other subtypes. At the two INF-α concentrations tested (26 pg/mL and 260 pg/mL), only IFN-α17 induced better stimulation of the Mx promoter than IFN-α2a did (p < 0.002 and p < 0.007 for 26 and 260 pg/mL, respectively).

**Figure 4:**
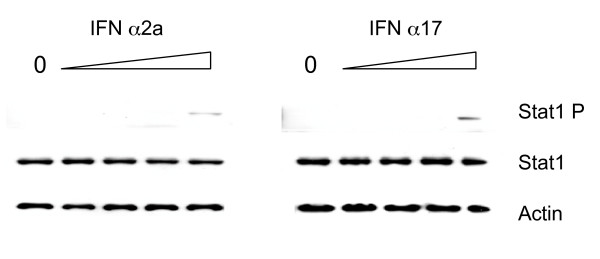
**Analysis of Stat1 phosphorylation**. A Western blot analysis was performed with monoclonal antibodies directed against specific tyrosine phosphorylation sites on Stat1 (Tyr701). The cells were treated with four concentrations of interferon (0.26, 2.6, 26 or 260 pg/ml) or not treated at all. The input control is represented by the Stat1 and the actin immunoblot.

In order to confirm the involvement of the Jak-Stat pathway, we analyzed the tyrosine phosphorylation status of the Stat1 protein. As shown in Figure 4, Stat1 phosphorylation was higher with IFN α17 than with IFN α2 at the same concentration. Altogether, these results suggest that IFN α17 is a stronger activator of the Jak-Stat pathway.

### Enhanced induction of antiviral proteins by IFNα-17

**Figure 5:**
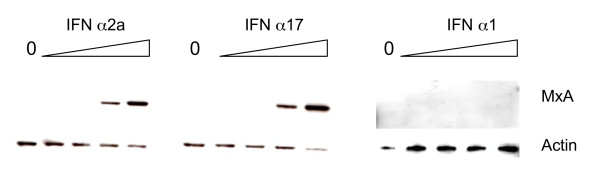
**Induction of MxA protein by IFN-α2a, 17 and 1**. Huh7 cells were treated with the same interferon concentrations as in Figure 4. The MxA protein was detected by immunoblotting. The input control is represented by the actin protein.

In order to explore the consequences of modulation of the Jak-Stat pathway by the different IFNα subtypes, IFN-regulated gene expression was explored using the TLDA. Comparative gene expression studies (IFN-α2a vs. IFN-α17 and IFN-α2a vs. IFN-α1) were carried out as described in the Materials and Methods section. As shown in Table 2, most of the genes induced by IFN-α17 treatment were well-characterized antiviral genes, such as the MxA, PKR, ADAR and OAS genes. Other highlighted genes (such as GBP1 or IFI27) may play a role in HCV replication [26,27]. Other genes have been described as being involved in interferon induction (DDX-58) or encoding proteasome subunits (PSMB8, PSMB9). Stronger expression of antiviral genes suggests stronger induction of the Jak-Stat pathway after IFN-α17 treatment. The above-mentioned genes were strongly less induced after treatment by IFN-α1 (up to 50-fold, see Table 2). To confirm these results, MxA protein expression was studied by Western blot analysis. As shown in Figure 5, MxA protein was not induced by IFN-α1 concentrations of 260 pg/ml or less. At equal IFN-α concentrations, MxA protein was more strongly induced by IFN-α17 than by IFN-α2a.

**Table 2:** ISG-RNA expression for IFN-α17 and IFN-α1, relative to IFN-α2a.

Detector	Alpha 17	Alpha1
18S-Hs99999901_s1	1.01	1.01
ADAR-Hs00241666_m1	0.99	0.80
ALCAM-Hs00233455_m1	0.90	1.05
APOL1-Hs00358603_g1	**2.28**	**0.26**
BAK1-Hs00832876_g1	0.86	0.95
CCL5-Hs00174575_m1	**1.44**	**0.07**
CD47-Hs00179953_m1	1.28	0.78
CXCL10-Hs00171042_m1	**1.42**	**0.43**
CXCL9-Hs00171065_m1	**5.89**	0.83
DDX58-Hs00204833_m1	1.28	**0.48**
EIF2AK2-Hs00169345_m1	0.92	**0.50**
FAS-Hs00236330_m1	0.89	**0.60**
FAS-Hs00531110_m1	0.48	**0.54**
G1P2-Hs00192713_m1	1.20	**0.23**
GAPDH-Hs99999905_m1	0.90	1.09
GBP1-Hs00266717_m1	1.15	**0.46**
GBP2-Hs00269759_m1	0.91	0.99
ICAM1-Hs00164932_m1	0.81	0.68
IFI27-Hs00271467_m1	**3.07**	**0.04**
IFI35-Hs00382709_m1	1.06	**0.64**
IFI35-Hs00413458_m1	1.09	**0.56**
IFI44-Hs00197427_m1	**1.72**	**0.50**
IFIH1-Hs00223420_m1	**1.37**	**0.21**
IFIT1-Hs00356631_g1	**1.32**	**0.22**
IFIT2-Hs00533665_m1	**1.36**	**0.75**
IFIT3-Hs00155468_m1	**1.46**	**0.41**
IFIT5-Hs00202721_m1	**1.39**	**0.42**
IFITM1-Hs00705137_s1	**1.37**	**0.56**
IFNAR1-Hs00265057_m1	0.90	1.10
IFNAR2-Hs00174198_m1	0.92	1.01
IL15-Hs00542571_m1	0.62	1.18
IL15RA-Hs00542604_m1	**6.85**	**2.38**
IL1B-Hs00174097_m1	1.18	0.71
IL6-Hs00174131_m1	0.97	**0.45**
IL8-Hs00174103_m1	1.28	0.89
IRF1-Hs00233698_m1	0.83	0.78
IRF2-Hs00180006_m1	0.94	1.09
IRF3-Hs00155574_m1	0.95	1.43
IRF4-Hs00180031_m1	0.85	0.61
IRF4-Hs00277069_m1	**4.35**	1.01
IRF5-Hs00158113_m1	1.05	**0.02**
IRF5-Hs00158114_m1	0.88	**0.17**
IRF8-Hs00609879_m1	0.59	**1.25**
ISG20-Hs00158122_m1	0.96	0.83
ISGF3G-Hs00196051_m1	0.96	0.51
MPO-Hs00165162_m1	1.24	0.84
MX1-Hs00182073_m1	**1.33**	**0.07**
MX2-Hs00159418_m1	1.01	1.16
MYD88-Hs00182082_m1	1.01	0.81
NFKBIA-Hs00153283_m1	0.85	0.93
NMI-Hs00190768_m1	1.18	0.80
OAS1-Hs00242943_m1	**1.38**	**0.15**
OAS2-Hs00159719_m1	**2.28**	**0.02**
OAS2-Hs00213443_m1	**2.72**	**0.03**
OAS3-Hs00196324_m1	1.18	**0.17**
OASL-Hs00388714_m1	**1.93**	**0.05**
PLSCR1-Hs00275514_m1	1.08	**0.58**
PML-Hs00231241_m1	1.17	0.80
PRKRA-Hs00269379_m1	0.96	1.13
PSMB10-Hs00160620_m1	1.08	0.93
PSMB8-Hs00188149_m1	1.86	0.92
PSMB8-Hs00544758_m1	**1.83**	**0.48**
PSMB9-Hs00160610_m1	0.96	2.44
PSMB9-Hs00544762_m1	**1.93**	**0.05**
PSMD8-Hs00601309_m1	1.06	1.14
PSME1-Hs00389209_m1	0.94	0.86
PTPRC-Hs00236304_m1	**0.56**	**1.39**
RELA-Hs00153294_m1	0.96	0.87
RELB-Hs00232399_m1	1.21	0.88
RSAD2-Hs00369813_m1	**2.17**	**0.12**
SP110-Hs00185406_m1	1.07	0.49
SP110-Hs00270142_m1	1.19	0.37
STAT1-Hs00234829_m1	1.03	0.53
STAT2-Hs00237139_m1	1.05	0.68
STAT3-Hs00374280_m1	0.93	1.02
STAT4-Hs00231372_m1	1.21	0.77
STAT5A-Hs00234181_m1	1.11	0.82
STAT5B-Hs00560035_m1	0.85	1.00
TNFSF10-Hs00234356_m1	0.98	0.82
TRADD-Hs00601065_g1	0.61	1.02
TRIM21-Hs00172616_m1	0.96	0.67
TRIM25-Hs00231947_m1	0.94	0.68
USP18-Hs00276441_m1	**1.24**	**0.62**

An ISG low-density array assay was performed with a concentration of 260 pg/mL of IFN. Indicated values are the mean of two independent experiments and represent the fold induction. The column named IFN-α17 corresponds to the comparison between IFN-α17 and IFN-α2a. The column named IFN-α1 corresponds to the comparison between IFN-α1 and IFN-α2a. Bold-faced values represent the genes strongly regulated by these two interferon compared to IFN-α2a. The detector column is the name of the gene follow by the Applied Biosytems references of the test.

## Discussion

Viral clearance in treated, chronically-infected HCV patients occurs in only about 55% of cases. At present, the standard therapy is a ribavirin-PEG-IFNα2 combination. Although the synergistic mechanism of action of this combination is not clearly understood [28], it is clear that therapeutic optimization is needed to increase the number of sustained virological responders. The aim of the present study was to determine the differential anti-HCV activity of the twelve main IFNα subtypes. We used the HCVcc system to study the subtypes' overall antiviral effects on the HCV life cycle.

We first tested the anti-HCV activity of the different IFNα subtypes by measuring the production of intracellular HCV RNA. Three subtypes (IFN α17, IFN α7 and IFN α8) were significantly more potent than IFN α2. These results are in accordance with other work demonstrating that IFN α8 has good antiviral activity against EMCV and HCV [16,19]. Little information on IFN α7 is available, although it displayed at least the same anti-HCV efficacy as IFN α2 in our study. This result was confirmed in terms of inhibition of the production of infectious particles, where IFN α17 was three times more potent than IFN α2. Again, this result agrees with previous work reporting that a variant of the IFN α17 subtype was more potent than IFN α2, although a comparison between the variant and the wild subtype was not presented [29]. Our study also confirmed the weak antiviral effect of IFN α1.

Concerning the mechanism of action, IFNα signaling is mainly related to the Jak-Stat pathway. Here, we sought to determine whether or not the higher activity of some subtypes was related to modified stimulation of the Jak-Stat pathway. As shown in the Results section, IFN α17 and IFN α7 prompted better stimulation of the ISRE-dependent genes than IFN α2 did at the same concentration. In contrast, IFN α1 was a poor activator of the Jak-Stat pathway. A Stat1 tyrosine phosphorylation study confirmed that the Jak-Stat pathway modulation was differentially modulated by the various different IFN α subtypes. This suggested that the Jak-Stat pathway was being modulated upstream of Stat phosphorylation. Recent work has detailed the interaction between IFN α2 and its receptor. IFN α2 binds first to IFNAR2 and then recruits IFNAR1. After formation of the ternary complex, the interferon signal is transduced via receptor-associated JAK kinases [30]. Three point mutations increase IFN α2's binding affinity for IFNAR1: H57A, E58A, Q61A. These residues were seen to be conserved in IFN α subtypes and were responsible for the differences in action between IFN β and IFN α [30]. Hence, it seems that modulation of IFN's affinity for the IFNAR1 induces an antiproliferative effect rather than modulating antiviral activity *per se *[30]. For IFNAR2, six hotspot residues for IFN α2's binding to IFNAR2 were highlighted (30, 33, 144, 147, 148 and 149) [31] but are conserved in all IFN α subtypes [32]. The weak effect of IFN α1 may be explained by the K31M mutation, which might disrupt the IFNα-IFNAR2 interaction [33]. A recent study of the interferon C-terminus domain has demonstrated that the tail residues are poorly conserved between the different IFN α subtypes and between IFN α and IFN β [34]. Moreover, major differences in the different tails' net charge were observed: IFN α2 has a net charge of 0 and IFN α8 and IFN α17 both have a net charge of 3. The replacement of IFN α2's tail by an IFN α8 tail increased the binding of IFN α2 to IFNAR2 by a factor of 20 and translated into nine-fold higher antiproliferative activity and four-fold higher antiviral activity [34]. These results are in agreement with our own, since IFN α17 and IFN α8 presented the greatest anti-HCV activity. Hence, it is possible that the differences between IFN α17, IFN α1 and IFN α2 in terms of antiviral activity could be due to differing affinities for the IFNAR2.

However, potential differences in affinity for the receptor cannot explain the totality of IFN α8's effect, where no significant increase in activation of the ISRE-dependent pathway was seen. Foster *et al*. have demonstrated that IFN α8 conserved this activity in U1A cells that do not contain Tyk2, a protein that is essential for IFN signal transduction [16]. Other pathways (such as the PI3K and p38 kinase pathways) have emerged as critical additional components of IFN-induced signal transduction [35]. For IFN α17, the increase in Jak-Stat signaling appears to be the main reason for its higher antiviral activity but stimulation of other pathways cannot be ruled out.

In addition, interferon's antiviral activity may depend on the cell type. In the present study, we used Huh7 cells, since these are the only currently permissive cell line for HCV replication in the HCVcc system. One could hypothesize that the difference in antiviral activity is explained by greater sensitivity of the Huh7 cells to IFN α17. However, very similar results were obtained with bovine Madin-Darby bovine kidney (MDBK) cells containing the chloramphenicol acetyl transferase (CAT) gene under the control of the MxA promoter [36]. It is difficult to say whether the activity differences between the IFN subtypes were due to the cell types or the viruses or both. However, we can hypothesize that the differences of activity are based on the virus type. For instance, the human MxA protein can induce protection against influenza virus or VSV [37]. PKR and 2-5A synthetases were both shown to be involved in resistance to EMCV but not VSV [38,39]. In this case, ISG induction could be modulated by the IFN subtypes and the transcriptome study may evidence modulation of antiviral-ISG expression and then subtype-specific modulation of the antiviral state. However, it is difficult to imagine how gene-by-gene modulation could be performed by induction of the Jak-stat pathway alone. Hence, cell type could be a factor – perhaps via modulation of the different IFN subtypes' affinity for the IFN receptor (as discussed above) or by stimulation of pathways other than Jak-Stat.

There are few available data on the production of IFN α17. In the context of HCV infection, IFN α5 is the major subtype produced in the liver and by the peripheral blood mononuclear cells (PBMCs) [35]. Hence, natural antiviral activity in the liver does not seem to be optimal in response to HCV infection, when combined with poor induction of the endogenous pathway [40].

## Conclusion

IFN α17 was the IFN α subtype that had the greatest anti-HCV activity in Huh7 cells. It was about three times more potent than the IFN α2 currently used in the clinic and this effect could be explained by stronger stimulation of the Jak-Stat pathway. We suggest that IFN α17 should be tested therapeutically with a view to improve treatment efficacy. It would also be interesting to test the synergy between IFN α17 and ribavirin.

## Competing interests

The authors declare that they have no competing interests.

## Authors' contributions

CFr and GD conceived, designed and wrote the paper. AD performed the analysis. VD and CFo gave their technical assistance for quantitative RT-PCR and virus assay. SC and CFr performed the statistical analysis. CW and JD have given final approval for the version to be published.
